# Genomic analysis of *Listeria monocytogenes* from US food processing environments reveals a high prevalence of QAC efflux genes but limited evidence of their contribution to environmental persistence

**DOI:** 10.1186/s12864-022-08695-2

**Published:** 2022-07-04

**Authors:** Devin Daeschel, James B. Pettengill, Yu Wang, Yi Chen, Marc Allard, Abigail B. Snyder

**Affiliations:** 1 Department of Food Science, Cornell University, Ithaca, NY USA; 2 Biostatistics and Bioinformatics Staff, Office of Analytics and Outreach, Center for Food Safety and Applied Nutrition, U.S. Food and Drug Administration, College Park, MD USA; 3 Division of Microbiology, Office of Regulatory Science, Center for Food Safety and Applied Nutrition, U.S. Food and Drug Administration, College Park, MD USA

**Keywords:** bcrABC, Comparative genomics, Persistence, Stress tolerance

## Abstract

**Background:**

Quaternary ammonium compound (QAC) efflux genes increase the minimum inhibitory concentration of *Listeria monocytogenes* (*Lm*) to benzalkonium chloride sanitizer, but the contribution of these genes to persistence in food processing environments is unclear. The goal of this study was to leverage genomic data and associated metadata for 4969 *Lm* isolates collected between 1999 and 2019 to: (1) evaluate the prevalence of QAC efflux genes among *Lm* isolates from diverse US food processors, (2) use comparative genomic analyses to assess confounding factors, such as clonal complex identity and stress tolerance genotypes, and (3) identify patterns in QAC efflux gene gain and loss among persistent clones within specific facilities over time.

**Results:**

The QAC efflux gene cassette *bcrABC* was present in nearly half (46%) of all isolates. QAC efflux gene prevalence among isolates was associated with clonal complex (𝛘^2^ < 0.001) and clonal complex was associated with the facility type (𝛘^2^ < 0.001). Consequently, changes in the prevalence of QAC efflux genes within individual facilities were generally attributable to changes in the prevalence of specific clonal complexes. Additionally, a GWAS and targeted BLAST search revealed that clonal complexes with a high prevalence of QAC efflux genes commonly possessed other stress tolerance genes. For example, a high prevalence of *bcrABC* in a clonal complex was significantly associated with the presence of the SSI-1 gene cluster (*p* < 0.05). QAC efflux gene gain and loss were both observed among persistent populations of *Lm* in individual facilities, suggesting a limited direct role for QAC efflux genes as predictors of persistence.

**Conclusion:**

This study suggests that although there is evidence that QAC efflux genes are part of a suite of adaptations common among *Lm* isolated from some food production environments, these genes may be neither sufficient nor necessary to enhance persistence. This is a crucial distinction for decision making in the food industry. For example, changes to sanitizer regimen targeting QAC tolerance would not address other contributing genetic or non-genetic factors, such as equipment hygienic design which physically mediates sanitizer exposure.

**Supplementary Information:**

The online version contains supplementary material available at 10.1186/s12864-022-08695-2.

## Background

Listeriosis is a global public health and economic burden. The etiological agent, *Listeria monocytogenes* (*Lm*), harbors in niches within food processing environments, resists removal through sanitation, and cross-contaminates food [1, 2]. Consequently, the persistence of *Lm* in food processing facilities is an important factor in outbreaks of listeriosis [1, 3–5]. QAC efflux genes have been proposed as contributors to environmental persistence [6]. QAC efflux genes increase the minimum inhibitory concentration (MIC) of *Lm* to benzalkonium chloride [7], but whether or not modest increases in MIC correspond to a practical increase in environmental persistence is less clearly established. Some studies have found an association between the presence of QAC efflux genes and the persistence of *Lm* in food facilities [7–10], but as many others have not found an association with QAC efflux genes or any other gene [11–13]. Similarly, other potential genetic determinants of persistence have been proposed, confounding associations with single genes [14].

The identification of *Lm* subtypes or genetic determinants associated with environmental persistence in food processing facilities has been the subject of numerous studies [14–17]. However, studies which only include isolates from an individual food processing facility cannot capture broad patterns in *Lm* ecology. By contrast, studies which exclusively analyze large datasets lack the resolution to assess nuances within specific environments. Here, we used both approaches to assess the role of genetic factors in *Lm* prevalence among food processing environments. The first objective of our study was to analyze a large (*n* = 4969) historical dataset of *Lm* collected from U.S. food processing facilities to identify patterns in *Lm* clonal complex and QAC efflux gene distribution. Analyses of this kind have only recently been enabled by the generation of large, open-source DNA sequence databases populated through food safety regulatory activities, the inclusion of sufficient metadata in those databases, and the availability of massively parallel bioinformatic tools [18, 19]. Comparison of our findings to similar, recent work from other countries [15, 20, 21] enabled our discussion of the global, genomic epidemiology of *Lm*. In addition to our comprehensive analysis, we analyzed changes in *Lm* populations within nine individual food facilities over time. We aimed to identify temporal changes in QAC efflux gene prevalence among clonal complexes (CC) across different food facility types. Overall, the goal of this study was to provide a comprehensive assessment of the QAC efflux gene patterns of *Lm* across U.S. food facilities and their relationship with environmental persistence.

## Materials and methods

### Isolate WGS data

**Fig. 1:**
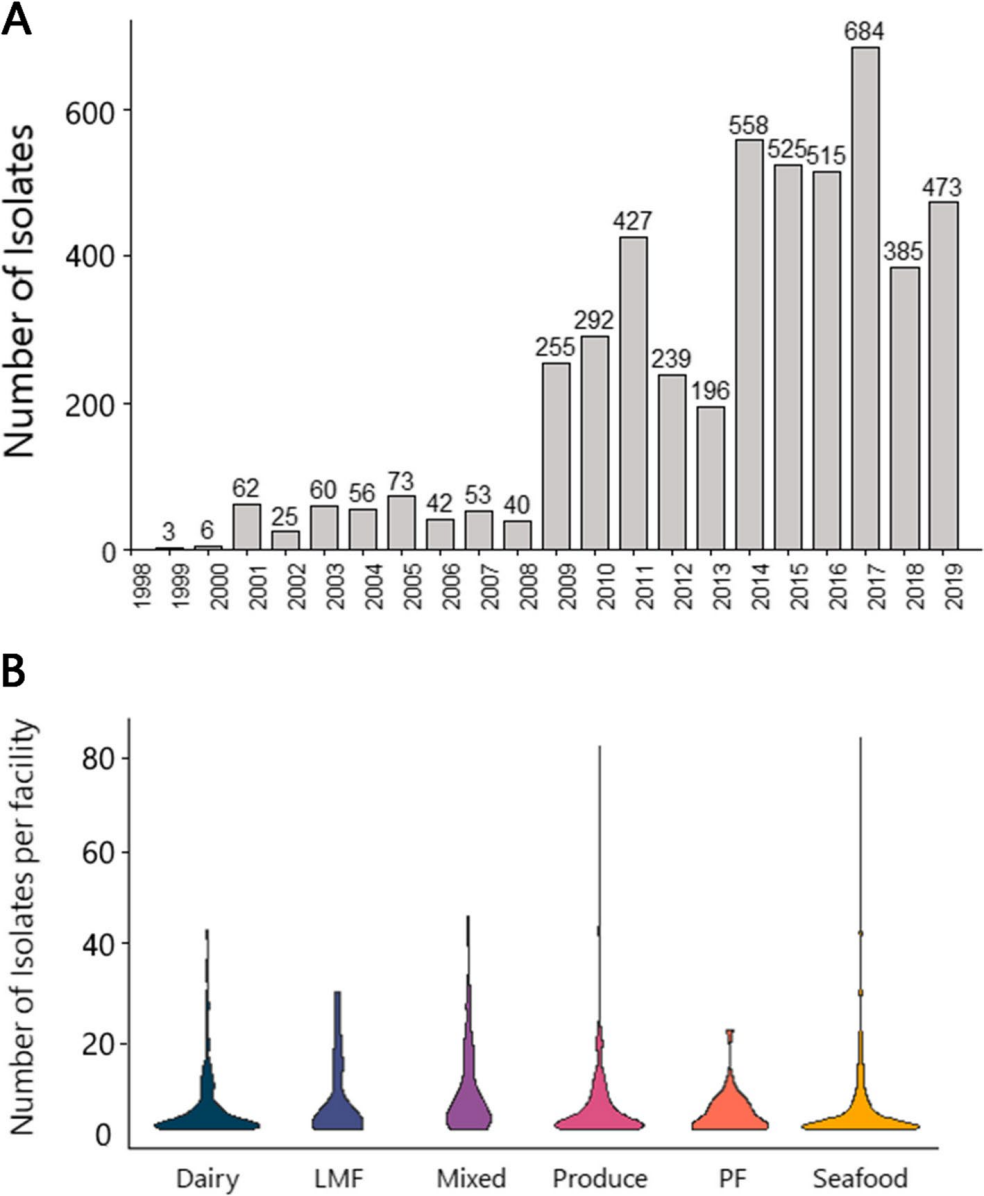
**A** Isolates were collected between 2009 and 2019. **B** Varying numbers of isolates were collected at each facility

A total of 4969 *Lm* isolates from food (*n* = 1223) and environmental swabs (*n* = 3746) were collected from June 1999 to November 2019 by the U.S. Food and Drug Administration’s (FDA) Center for Food Safety and Applied Nutrition (CFSAN) as part of typical agency surveillance and investigative activities (Fig. 1A). No clinical specimens were included in this collection. Raw reads were downloaded from the NCBI Sequence Read Archive database using a list of all the NCBI Pathogen Database Biosample accession numbers that 1) had linked metadata, described below, and 2) were assigned a “*Listeria monocytogenes*” organism identification tag. Sequence Read Run codes for all isolates used in this study are provided in Additional file 1. Reads were downloaded in bulk with a custom Bash script and stored on the Cornell Institute of Biotechnology’s cloud computational platform (BioHPC) for further analysis. Reads for all 4969 *L. monocytogenes* isolates were trimmed using BBDuk from the BBMap package v38.90 to remove adapter sequences leftover from Next Generation Sequencing. Trimmed reads were then assembled into draft genomes in parallel using SPAdes v3.15.2 [22] and GNU Parallel v20170522 [23]. Draft genomes were then quality checked using QUAST v5.1.0rc1 [24].

### Source attribution metadata

Metadata for each isolate included the collection date, product description, and an anonymous code for the responsible firm. Metadata extraction and cleaning from the FDA Field Accomplishments and Compliance Tracking System (FACTS) database required extensive curation as described previously by Wang et al. [25] The identities of food facilities were anonymized. The product description feature was populated from an open-response field completed by the data collector. Due to the subsequent variation in the detail provided from these unstructured responses, the facility descriptions were condensed into six categorical types based on the commodities handled, as follows: seafood (*n* = 209 facilities), produce (*n* = 196), mixed (*n* = 157), dairy (*n* = 151), pet food (PF) (*n* = 24) and low moisture foods (LMF) (*n* = 15). Mixed facilities included various RTE foods such as pre-made wet salads, deli sandwiches, spreads, and frozen pizzas. Although there are hypothetical cases where multiple facilities could be associated with a given contaminated product as it moves throughout the supply chain, only isolates with a single responsible facility identified (i.e. source of the contaminant) were recorded.

### BLAST screens

A custom BlastP search was used to screen each isolate for the presence or absence of QAC efflux genes *bcrABC*, *qacH*, *emrC*, and *emrE* as well as various stress response genes. Reference sequences for multiple alleles of each QAC efflux gene were collected from PubMLST’s Bacterial Isolate Genome Sequence Database (BIGSdb), subsection BIGSdb-*Lm* hosted by the Pasteur Institute [26]. Reference sequences from the BIGSdb were accessed September 9th, 2020. The BIGSdb grouped alleles of *qacH* and *emrC* together under the name Tn6188_qac (emrC) despite significant differences in sequence identity between these genes [27]. The BIGSdb sequences for *emrC* (allele ID 1 and 7) and *qacH* (allele ID 2–6 and 8–9) were segregated in our reference collection. Sequences for stress response genes were taken from the literature review of Pasquali et al. [28]. BlastP searches were performed with a cutoff of ≥90% sequence identity and ≥ 50% sequence coverage. Hits for *bcrABC* were cross checked using AMRFinderPlus [29] according to default parameters with a cutoff of ≥90% sequence identity and ≥ 50% sequence coverage. Isolates were also screened using a BlastN search to detect the presence of *inlA* genes with a premature stop codon (PMSC). The *inlA* allele database from BIGSdb was downloaded and used in the BlastN search. Isolates were marked as having a PMSC *inlA* gene if they had a BlastN hit with 100% sequence identity and 100% sequence coverage to an *inlA* allele from the BIGSdb flagged as containing a PMSC.

### Clonal complex assignment

Isolates were assigned to a clonal complex based on a seven gene multilocus sequence type (MLST) scheme [30]. In short, reference allele sequences and clonal complex profiles were collected from the BIGSdb accessed on March 8th, 2021, with 2672 allele profiles available at that time. Isolate assemblies were blasted against this database of reference allele sequences using BlastN. A custom R script was used to filter for allele hits with 100% nucleotide identity and 100% coverage. These hits were then added to the MLST profile for each isolate and clonal complexes were assigned based on completed seven allele profiles.

### Computation of minimum SNP distances

The dataset was filtered for facilities with at least 20 isolates collected across at least four sampling point years. Nine facilities matched these criteria: four seafood (facilities A-D), three mixed (facilities E-G), one dairy (facility H) and one produce (facility I). Read coverage for each isolate was checked by comparing the raw read files (fastq) and the completed assembly (fasta) using a custom bash script. Isolates with poor read coverage (< 30) or poor assembly metrics (QUAST v5.0.2) indicating contamination or fragmentation were excluded from the analysis. Pairwise SNP distances were calculated among isolates of the same clonal complex within each facility using the CFSAN SNP Pipeline v2.2.1 [31]. The highest quality isolate based on genome length, GC content, and assembly fragmentation (N50) was chosen as the reference assembly for each group. The metrics.tsv output file from the pipeline was used to check for isolates with a low percentage of mapped reads and these isolates were excluded from downstream analysis.

### Genome-wide association study

Isolates were classified as either containing (*n* = 2474) or not containing (*n* = 2495) a QAC efflux gene (*bcrABC*, *qacH*, or *emrE*) for the purpose of a genome-wide association study (GWAS) to identify other genes that were associated with the presence of QAC efflux genes. The genomes were annotated using Prokka v1.14.5 [32] and error corrected using Panaroo v1.2.8 [33]. Statistical associations were calculated using Scoary v1.6.14 [34]. Scoary results were filtered for results with a gene ID in the UniProt database and having a “worst” pairwise comparison *p*-value < 0.001 [35].

### Statistical analysis and data visualization

**Fig. 2:**
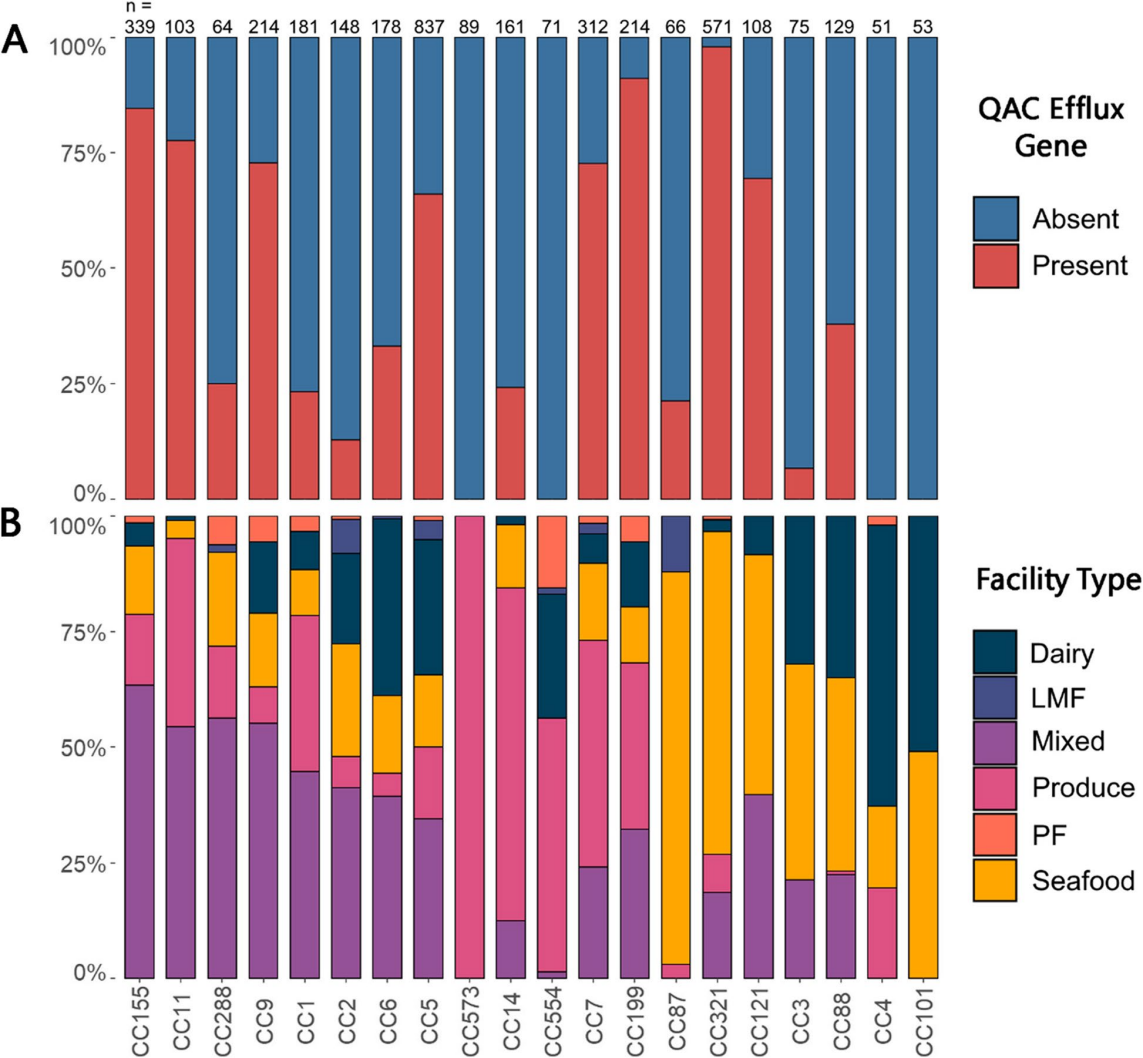
**A** QAC efflux genes were unevenly distributed among clonal complexes. **B** Clonal complexes were associated with different facility types. The 20 most common clonal complexes are presented and the number of isolates for each clonal complex (n) is noted at the top of the figure. Clonal complexes are clustered by the facility type that had the most isolates for each clonal complex

**Fig. 3:**
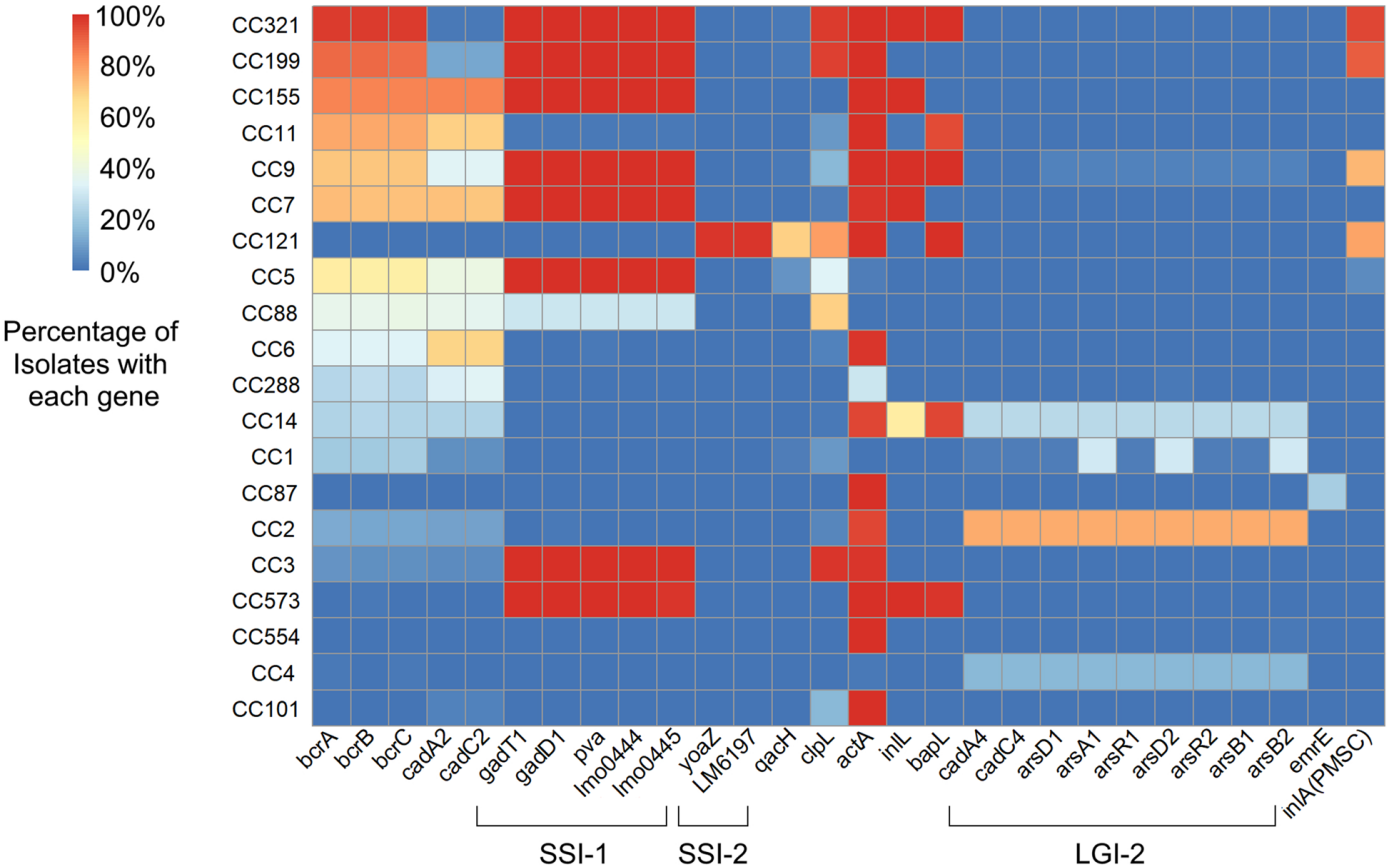
The prevalence of environmental stress tolerance genes among clonal complexes with high prevalence of QAC efflux genes. Clonal complexes are ordered by the prevalence of QAC efflux genes among their isolates

Statistical tests and graph creation was done in R version v4.1.0. Logistic regression was performed using the generalized linear model (glm) function and pairwise comparisons were evaluated using the emmeans function from the emmeans package v1.6.2–1 with the Tukey method of p-value adjustment for multiple comparisons. Logistic regression was used to determine if the type of facility an isolate was collected from was a significant predictor of the presence or absence of QAC efflux genes. Linear regression with the linear model (lm) function was used to check whether the prevalence of QAC efflux genes in a clonal complex was a significant predictor of the presence of various stress genes. Chi-squared tests with FDR correction for multiple hypothesis testing were used to check for an association between each clonal complex and the type of facility it was isolated from. A chi-squared test was also used to check for an association between clonal complex and the prevalence of QAC efflux genes. The following R packages were utilized for making figures: ggplot2 version 3.3.5 (Fig. 1A, B, Fig. 2, Fig. 3), pheatmap version1.0.12 (Fig. 1C), and packcircles version 0.3.4 (Fig. 3).

## Results/discussion

### QAC efflux genes were detected in half of all isolates and were associated with specific clonal complexes

Isolates were collected from 752 unique U.S. food processors and the number of isolates collected each year varied, increasing with the growing application of genomics to food safety surveillance within the past decade (Fig. 1A). Each facility had at least one *Lm* isolate; however, the total number of isolates collected from each facility ranged from 1 to 84 (Fig. 1B). In total, 28% of facilities had only one *Lm* isolate across their collection history and 82% of facilities had fewer than 10 isolates. Facilities with more than 20 isolates accounted for only 7% of all facilities. Out of all isolates, 50% contained one or more of the genes *bcrABC*, *qacH*, or *emrE*. This corresponded to 354 facilities (47%) where at least one *Lm* isolate with a QAC efflux gene was detected. Isolates containing the *bcrABC* QAC efflux cassette (2300, 46%) were found in 318 facilities (42%) and accounted for the majority of QAC efflux genes detected. Isolates carrying *qacH* (170, 3%) were found in only 54 facilities (7%) and isolates with *emrE* (20, 0.4%) were in only 6 facilities (0.8%). Nearly half (76/170) of *qacH* containing isolates came from three facilities: a dairy facility producing cheese spreads, a mixed facility producing ready-to-eat sandwiches, and a seafood facility producing smoked fish. The majority of *emrE* containing isolates (14/20) came from a single seafood facility. The *emrC* gene was not found in any isolate within this collection.

The 20 most abundant clonal complexes accounted for 80% of all isolates in our collection (Fig. 2) and ranged from CC5 (837, 17%) to CC4 (51, 1%). All 20 of the most abundant clonal complexes were found to a have significant association with facility type (𝛘^2^ < 0.001). For example, while CC5 was one of the more evenly distributed clonal complexes, a chi-squared test of association corrected for multiple hypothesis testing indicated that CC5 was overrepresented in mixed and dairy facilities (Fig. 2B). Some clonal complexes were clearly isolated primarily from a single facility type, such as CC573 and CC14 from produce facilities and CC87 and CC321 from seafood facilities. QAC efflux gene prevalence was associated with clonal complex type (𝛘^2^ < 0.001) and varied from 0 to 98% prevalence. For example, CC573, CC554, CC4 and CC101 did not have a single isolate with a QAC efflux gene. By contrast, other clonal complexes had high rates of *bcrABC* presence such as CC321 (98%), CC199 (89%), and CC155 (84%).

In comparison with other recent large-scale studies, our results suggest that there are regional differences in the prevalence of QAC efflux genes and clonal complexes. We found that *bcrABC* was present in 46% of isolates from US food processors. Cooper et al. [20] analyzed 1279 *Lm* isolates from Canadian foods and food processing environments and found that 59% contained *bcrABC*, suggesting a similar rate between Canadian and U.S. food facilities. In contrast, recent European studies have reported a much lower prevalence of *bcrABC* in *Lm* isolates. Painset et al. [21] analyzed 1144 *Lm* isolates associated with RTE foods from EU member nations and found that *bcrABC* was only detected in 5% of isolates. Maury et al. [15] analyzed 2982 food and clinical *Lm* isolates from France and found that *bcrABC* was present in only 8% of isolates. Interestingly, the prevalence of the *qacH* efflux pump also appears to differ by region. In our study, *qacH* was present in 5% of isolates, and in the Canadian study it was present in only 1% of isolates, yet the EU study and the French study both reported *qacH* in 19% of isolates.

These regional differences in QAC efflux gene prevalence are possibly associated with regional differences in clonal complex prevalence. The six most abundant clonal complexes in our study were CC5 (17%), CC321 (11%), CC155 (7%), CC7 (6%), CC9 (4%), and CC199 (4%). These findings are concordant with the Canadian study which reported CC5, CC321, CC155, and CC7 among their most abundant clonal complexes [20]. We found that these clonal complexes have a high prevalence of *bcrABC*, as high as 98% in the case of CC321 (Fig. 2A). Some of these clonal complexes, most notably CC321, were comparatively rare in the EU and French studies. By contrast, clonal complexes that have a high prevalence of *qacH,* such as CC121, were more common in the EU and French studies than in our study and the Canadian study. CC121 was the most abundant clonal complex identified in the French collection, but more than half of their CC121 isolates were from meat facilities [15], which may explain the diminished prevalence in our collection as the FDA does not regulate meat facilities. Only 108 CC121 isolates were identified in our study and 52% were from seafood facilities. These CC121 isolates represented only 4.5% of all seafood associated isolates collected here compared to the collection from France in which CC121 accounted for about half of all seafood associated isolates and similarly in the EU study where CC121 isolates comprised more than half of all isolates from fish and fishery products. Notably, differences in QAC sanitizer usage between regions could potentially affect the prevalence of QAC efflux genes in a region. The EU passed a law in 2014 which limited the acceptable amount of residual benzalkonium chloride on food products which has discouraged their use in the food industry [36]. Indeed, lack of generalizable metadata on sanitizer usage among different industries, regions, and individual facilities complicates all analyses investigating QAC efflux gene proliferation. The prevalence of *emrE* between regions also varied. Cooper et al. [20] detected *emrE* in 6% of Canadian isolates which was higher than the current study and the European studies which all found *emrE* in less than 0.5% of isolates. Indeed, the original paper on *emrE* concerned a listeriosis outbreak linked to sliced meat which killed 22 people in Canada in 2008 [6]. One study found LGI1, the genomic island that carries *emrE*, in 88% of Canadian clinical isolates that belonged to CC8, a clonal complex highly associated with listeriosis in Canada [37]. CC8 was also the third most common clonal complex in the Canadian study by Cooper et al. [20] which may explain the increased prevalence of *emrE* in that region.

### Associations between QAC efflux genes and other stress adaptations complicates conclusions on the direct role of QAC efflux genes in *Lm* environmental persistence

We investigated whether clonal complexes with a high prevalence of QAC efflux genes were more likely to carry other stress tolerance genes by conducting a GWAS (Table 1) and a targeted BlastN search (Fig. 3). GWAS analysis identified several genes associated (*p* < 0.0001) with the presence of QAC efflux genes that are putatively involved in either DNA replication or stress response (Table 1). The gene names in Table 1 are based on the annotation software’s (Prokka) protein sequence database and it should be noted that some gene names may differ in *Lm*. For example, *ebrB* and *qacC* identified in Table 1 are likely *bcrC* and *bcrB,* respectively. Since *bcrABC* is plasmid encoded, we expected some of the associated genes identified in this GWAS to be carried on the same plasmid. In fact, our analysis identified the cadmium stress response genes *cadA2*/*cadC2* which are frequently contained on the same composite transposon as *bcrABC* [38]. Genes involved in replication such as *slmA* may also be associated with plasmid function. Interestingly, DNA Polymerase IV was found to be associated with QAC efflux genes and this gene is known to play a role in the SOS response. One associated gene, *cwlO*, is related to peptidoglycan synthesis. Peptidoglycan synthesis has previously been suggested to contribute to QAC tolerance by limiting degradation of the cell wall [8]. Genes involved in the stress response to copper, heat, and mercury were also associated with QAC efflux genes, though the latter was found in only a small number of isolates (approximately 5%) and may simply be associated with specific clonal complexes that are likely to possess *bcrABC*.

**Table 1:** Genes involved in DNA replication, recombination, and stress response were commonly associated with the presence QAC efflux genes

Gene	Gene Function	UniProt ID	Positive isolates with a QAC efflux gene (n/2490)	Positive isolates without a QAC efflux gene (n/2479)
*hin*	DNA invertase	P03013	2175	2
*ebrB*	Multidrug efflux pump	P0CW83	2283	2
*copY*	Transcriptional repressor in response to copper	Q47839	1138	17
*slmA*	Nucleoid occlusion factor	P0C093	2281	1
*nucH*	Thermonuclease	P43270	1051	14
*rapA* / *dbpA*	Stimulates RNA polymerase recycling in stress conditions / ATP-dependent RNA helicase	P60240 / Q814I2	1168	25
*parA* / s*oj*	Chromosome partitioning / Chromosome partitioning ATPase	B8GW31 / Q72H90	1693	162
*ravA*	ATPase possibly involved in cadmium stress response	P31473	1192	0
*bin*	Tn522 DNA invertase	P20384	1264	120
*qacC*	Multidrug efflux pump	P14319	2290	1
*cwlO*	Peptidoglycan endopeptidase	P40767	1044	14
*qorB*	Quinone oxidoreductase	P39315	1390	5
*cadA*	Cadmium transporting ATPase	P20021	1212	141
*cadC*	Cadmium resistance transcriptional regulatory protein	P20047	1192	141
*dinB*	DNA polymerase IV	Q47155	1125	164
*clpB*	Chaperone protein involved in heat stress response	P53532	664	0
*merB*	Alkylmercury lyase	P77072	190	0
*merR*	Mercuric resistance operon regulatory protein	P22853	190	1
*cueR*	Transcriptional regulator involved in copper response	P0A9G4	193	2
*hin*	DNA invertase	P03013	187	1
*merA*	Resistance to mercury	P17239	191	2

For the targeted BlastN search we drew from a list of environmental stress response genes curated by Pasquali et al. [28]; however, this list is not comprehensive and does not cover all known stress response genes in *Lm*. We found that clonal complexes with a higher prevalence of *bcrABC* were more likely to carry the stress tolerance islet SSI-1 based on linear regression (*p* < 0.05), but no other gene had a similar association (Fig. 3). Still, our analysis showed that there are numerous clonal complexes that appear to have a high prevalence of both QAC efflux genes and stress genes (Fig. 3). Maury et al. [15] found that clonal complexes with a truncated *inlA* gene (a marker for hypovirulence in *Lm*) had more stress adaptations and were more common among food or environmental isolates, and that clonal complexes with a complete *inlA* gene had less stress resistance and were common among clinical isolates. This suggests that some clonal complexes appeared to be stress adapted, others host adapted, while others were between those two extremes [39]. Indeed, through our BlastN search we found that only four clonal complexes (CC321, CC199, CC9 and CC121) frequently had a truncated *inlA* gene and that all these clonal complexes also contained QAC efflux genes and other non-core stress response genes at a high frequency (Fig. 3). Interestingly, Mahoney et al. [39] found that *Lm* isolates with a truncated *inlA* gene were associated with an increased cold adhesion phenotype, suggesting that there is an evolutionary tradeoff between virulence and adhesion with respect to truncations in *inlA*. Therefore, truncations in *inlA* may itself be a stress adaptation. Overall, our results suggest that clonal complexes which contain QAC efflux genes may be more likely to harbor other stress response genes. This complicates the direct evaluation of the effect of QAC efflux genes on *Lm* persistence in food facilities since clonal complexes associated with QAC efflux genes may differ in many genetic attributes that impact stress tolerance and persistence.

### *Lm* isolates from mixed and seafood processing facilities were most likely to have a QAC efflux gene

**Fig. 4:**
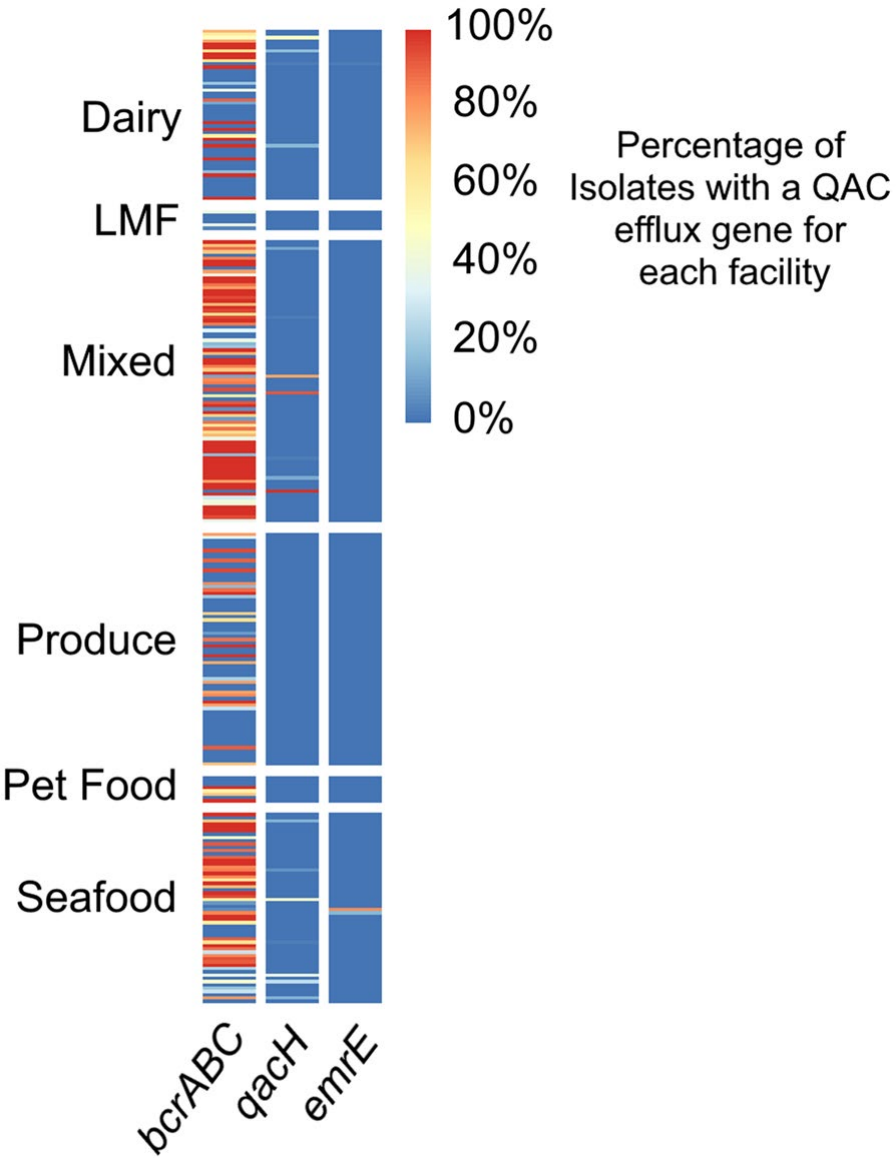
The isolates from mixed and seafood facilities more often contained a QAC tolerance gene compared to other food facility types. Each band on the intensity map represents an individual facility (*n* = 281) that had at least 5 isolates in its collection history

The proportion of *Lm* isolates with a QAC efflux gene varied from 0 to 100% among facilities that had at least five *Lm* isolates in its collection history (Fig. 4). Overall, isolates from mixed facilities had the highest prevalence of QAC efflux genes (75%) followed by isolates from seafood facility isolates (67%), though this was a significantly lower level (*p* < 0.001). Isolates from dairy (34%), produce (32%), pet food (29%), and LMF (5%) food handling facilities all had significantly lower rates of QAC efflux gene presence compared to either mixed or seafood handling facilities (*p* < 0.001). Although most QAC efflux genes were *bcrABC*, these observations were also true for *qacH* when evaluated independently (Fig. 4). The association of QAC efflux genes with facility type has been previously reported. For example, a high rate of QAC efflux gene detection in isolates from meat facilities has been reported. Cooper et al. [20] found that QAC efflux gene prevalence was lower in produce associated isolates than among isolates collected from facilities which handle animal products, and, more specifically, in meat associated isolates. Meat isolates were not represented in our study because those facilities are not regulated by the FDA; however, we did find a lower prevalence of QAC efflux genes in produce when compared to seafood. Additionally, we observed a high level of QAC efflux genes among isolates from mixed facilities which includes products that may contain meat content below 3% (raw) or 2% (cooked) in their ingredients. Maury et al. [15] similarly reported that QAC efflux genes and other stress tolerance genes were associated with isolates from meat products.

The prevalence of specific clonal complexes in different facility types likely influences the prevalence of QAC efflux genes in those facilities. For example, we found CC321 to be highly associated with seafood facilities and we found that CC321 isolates have *bcrABC* genes at an extremely high rate (Fig. 2A). Numerous other studies have also reported CC321 in association with meat, poultry, and seafood processing operations [19, 20, 40]. Therefore, our finding that QAC efflux genes were more common in seafood facility isolates can be partially explained by the high frequency of CC321 isolates from seafood facilities. Similarly, Maury et al. [15] also found that isolates from seafood and meat facilities were commonly identified as CC9 and CC121 which also contain a high prevalence of QAC efflux genes. The association of clonal complex with certain environments may be attributable to broader stress resistance genotypes. For example, CC321 and CC9 frequently contained *bcrABC* but also frequently contained the SSI-1 stress islet as well as genes related to surface adhesion such as *bapL* and a truncated *inlA* (Fig. 3). Previous studies have identified CC321 and CC9 as stress adapted, environment specific clonal complexes [15, 41, 42].

There is also evidence that *Lm* isolated from natural environments may be comparatively less adapted to the stresses of food processing environments. One study found that *Lm* isolated from animals and the natural environment were more susceptible to QACs and peracetic acid than *Lm* from food [43]. Liao et al. [44] collected 177 *Lm* isolates from natural soil environments across the US. We found that none these 177 isolates contained a QAC efflux gene and only 64 of the isolates could be assigned a clonal complex based on existing MLST profiles in the BIGSdb. Of those 64, only eight isolates were assigned to clonal complexes that were among the 20 most abundant clonal complexes from our collection of food product and food processing environment isolates. Additionally, these eight isolates were from CC4, CC554, CC1, and CC6 which all have a low prevalence of QAC efflux and stress response genes (Fig. 3). This suggests that *Lm* from the natural environment may be less likely to have a stress resistance genotype compared to *Lm* collected from food processing environments. This is aligned with our observation that QAC efflux genes are part of a suite of adaptations associated with specific clonal complexes common among *Lm* isolated from some food production environments.

Differences in environmental conditions between facility types should also be considered. There are some commodity-specific trends in sanitizer use. For example, the meat and dairy industries commonly use chlorinated sanitizers while produce facilities more commonly use peroxyacetic acid. Dairy processors may also avoid using QACs over concerns that residuals might harm cheese manufacturing [45]. And in some facilities QACs may only be employed for the decontamination of footwear rather than food contact surfaces [46]. Differences in harborage sites or sanitation operations could also potentially create variable selective pressures between different types of food facilities. Alternatively, several possible factors beyond selection pressures may contribute to differences in clonal complex diversity within a facility. The raw materials or the regional environment may influence the unique ecology of *Lm* in a given facility [44, 47]. A longitudinal study of Finnish dairy cattle farms found related genotypes in the milk processing facility and the outside farm environment, suggesting a relationship between the raw material and the production environment [48]. Another study traced *Lm* contamination of a slaughterhouse back to genotypes on incoming pigs [49]. Additionally, the initial *Lm* to colonize a facility can influence the long-term diversity of clonal complexes in the facility. The effect of microbial founder species on microbial succession and ecology of an environment remains under researched, especially in food processing environments [50]. One study that monitored the colonization dynamics of a new meat processing facility found that a persistent pulsotype of *Lm* had colonized the facility in less 6 months, and was identical to a pulsotype found at a raw ingredient provider [51]. Similarly, a new cheese processing plant did not detect *Lm* in the facility for the first 9 months of operations, and then detected a new strain that persisted and spread throughout the facility [41]. The microbiome of a food processing facility may also influence the composition of the *Lm* populations in a facility. Past research has shown that the presence of *Pseudomonas putida* biofilms can increase the attachment of *Lm* [52, 53] and that native microbiota in fruit processing plants were associated with presence of *Lm* [54]. Collectively, although there is strong evidence of associations among clonal complex, facility type, and the presence of stress tolerance genes, the specific driving dynamics behind these associations are complex and may be multifaceted.

### Analysis of individual facilities with large collection histories reveals evidence of persistent *Lm* but limited association with QAC efflux genes

Evidence of persistent *Lm* was identified in all nine individually analyzed facilities. Although there is not a single consensus definition for *Lm* persistence, in general, persistence refers to the repeated isolation of genetically related *Lm* across a given time period in a given food processing environment [55, 56]. More liberal definitions of persistence consider time periods as short as 3 months to be evidence of persistence [9], whereas more conservative definitions require repeated isolation over time periods as long as 16 months [10]. Besides duration between isolation date, the second criterion for persistence is the cutoff for “genetically related.” A common threshold used in WGS analysis is a SNP distance of ≤20 between two isolates originating from a common source [57]. For this analysis, we considered groups of isolates collected in different years from the same facility with a mean SNP distance ≤20 to be evidence of persistent *Lm*. However, the possibility that repeated isolation events were due to re-introduction of clones to the processing facility from contaminated ingredients rather than persistence of isolates within the processing environment cannot be excluded. Under this definition, all nine facilities had evidence of persistent *Lm* (Table 2). Persistent isolates were collected up to 17 years apart in the case of seafood facility D in which there was a CC321 product isolate from 2000 that was 14 SNPs different from an environmental CC321 isolate from 2017. These findings are consistent with other studies that have documented closely related *Lm* isolates surviving in food processing environments for extended periods [8, 58–60].

**Table 2:** All 9 individually analyzed facilities had evidence of persistent *Lm*. Groups of *Lm* from each facility are organized by clonal complex and in some cases group number. I.e. in facility B there were two genetically distinct groups of CC321 isolates based on SNP distance

Facility CC (group #)	Collection Period	Mean (Max) Pairwise SNPs	Isolates (n)	***bcrABC*** (n)	***qacH*** (n)
Facility A CC6	2009–2016	3.7 (6)	14	0	0
Facility A ST1048	2016–2019	4.7 (7)	4	0	0
Facility B CC321 (1)	2013–2018	1.3 (3)	14	14	0
Facility B CC321 (2)	2011–2013	3 (5)	3	3	0
Facility C CC321	2011–2017	7.1 (18)	47	47	9
Facility C CC5	2004–2017	14 (28)	18	0	17
Facility D CC155	2011–2014	10 (17)	7	7	0
Facility D CC199	2011–2014	11.8 (19)	4	4	0
Facility D CC321	2000–2017	13.7 (23)	20	20	0
Facility E CC6	2011–2013	5.1 (11)	9	1	0
Facility E CC155	2011–2017	10.8 (21)	15	13	0
Facility F CC6	2015–2018	11 (20)	13	13	0
Facility G CC11	2012–2019	7.8 (16)	10	10	0
Facility H CC5	2016–2019	4.1 (7)	11	3	0
Facility I ST2629	2008–2012	6.2 (15)	43	0	0

**Fig. 5:**
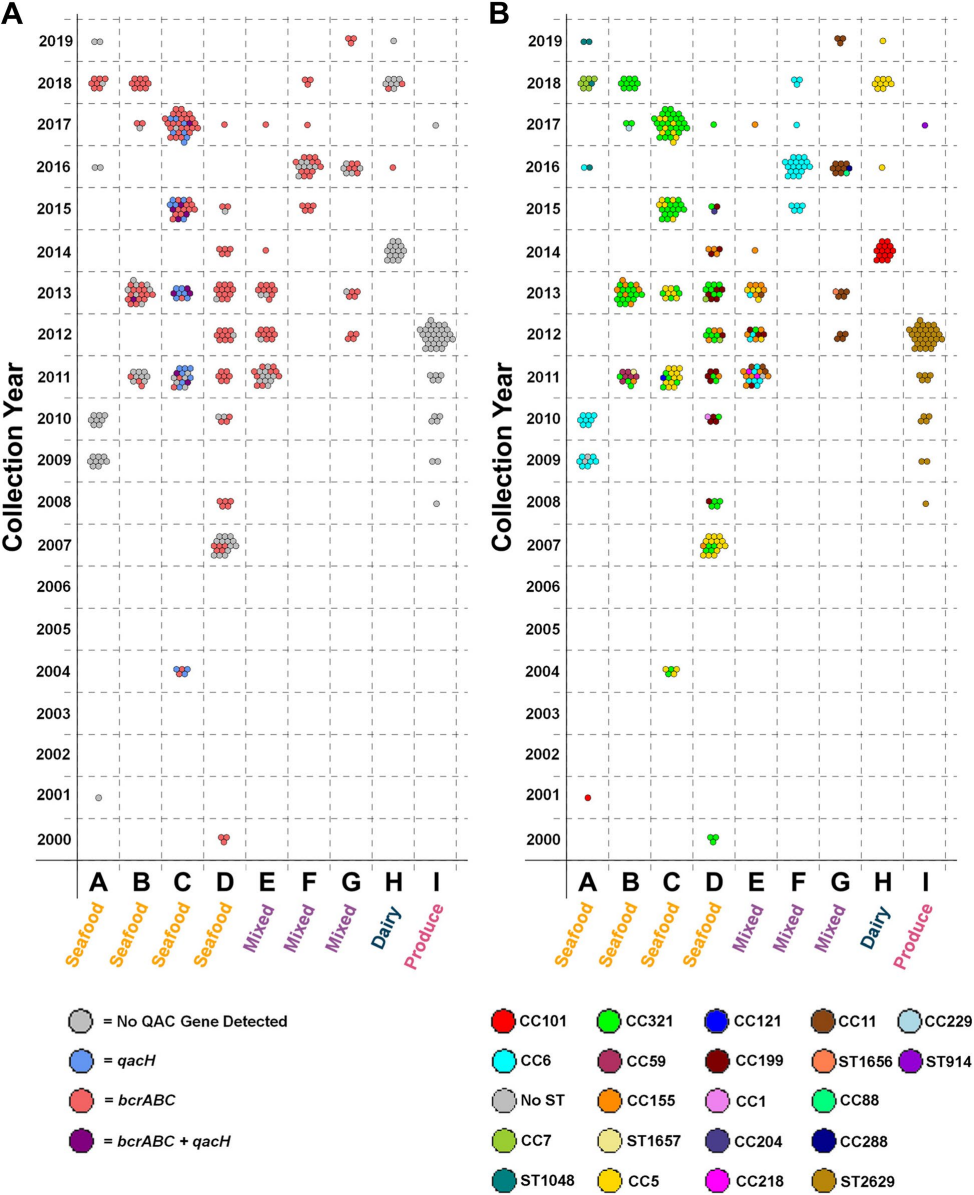
The prevalence of QAC efflux genes (**A**) and clonal complexes (**B**) varied across time in different food facilities. Each point represents a unique Lm isolate collected in the given year and facility. Points on Fig. 3A and B are mirrored and correspond to the same isolate

It has been suggested that QAC efflux genes contribute to persistence [1, 7, 9, 10, 14, 20]. Notably, the lack of metadata documenting the use of QAC sanitizers within individual facilities limits our assessment of this issue, but our analysis did reveal inconsistent patterns in QAC gene prevalence over time even within the same facility. Importantly, regulatory environmental sampling is potentially biased because it is not designed as a survey but is instead investigatory, and we have limited knowledge of the sampling structure and collection strategy. Acknowledging these limitations, we defined prevalence as the proportion of isolates from a given genotype out of all positive isolates over the collection history of a facility. In some cases, the prevalence of isolates with QAC efflux genes appeared to increase over the time. For example, seafood facilities B, C, and D all contained persistent CC321 populations that increased in prevalence over time, and every CC321 isolate had *bcrABC* (Fig. 5)*.* Concurrently, isolates of other clonal complexes decreased in prevalence over time. For example, in seafood facility B, CC59 isolates which all lacked a QAC efflux gene were the most prevalent clonal complex in 2011 (6/11) but were never isolated again after 2011. Similarly, in seafood facility D, CC5 isolates which all lacked QAC efflux genes were the most prevalent clonal complex in 2007 (13/18) but were not isolated in later years. These examples show how changes in the prevalence of QAC efflux genes within facilities were generally linked with changes in the prevalence of specific clonal complexes. This confounds assessments on the role of QAC efflux genes as clonal complexes possess many genetic differences that may contribute to persistence. For example, in seafood facility A all instances of a QAC efflux genes were from isolates of CC7 (Fig. 5). And in seafood facility D all instances of a QAC efflux gene were from isolates of CC321. Therefore, in seafood facility D as the prevalence of CC321 strains rose from 25% in 2011 to 76% in 2017 so did the prevalence of QAC efflux genes (Fig. 5). By contrast, there were a few examples of specific clonal complexes with variable QAC efflux gene presence among isolates. In 2016 in mixed-type facility F there were CC6 isolates both with (*n* = 12) and without (*n* = 7) *bcrABC*, but by 2018 only CC6 isolates with *bcrABC* (*n* = 3) were detected. This was also observed in mixed facility G where in 2016 there were CC11 isolates both with (*n* = 4) and without (*n* = 4) *bcrABC* but by 2019 only CC11 isolates with *bcrABC* (*n* = 3) were detected. This represents some of the most compelling evidence that QAC efflux genes contributed to persistence in food processing environments; however, these findings were not universal particularly as we increased the resolution of our assessment.

Beyond the overall prevalence of QAC efflux genes among all *Lm* within a facility, we also tracked persistent isolates of closely related *Lm* and evaluated patterns in the presence of QAC efflux genes. For example, in produce facility I there was a population of persistent ST2629 isolates with a mean SNP distance of 6.2 collected between 2008 (*n* = 1) and 2012 (*n* = 31) that did not contain QAC efflux genes and became more prevalent over time. Similarly, there were persistent CC6 isolates with a mean SNP distance of 3.7 collected between 2009 (*n* = 7) and 2016 (*n* = 1) that did not have QAC efflux genes in seafood facility A. In mixed facility E there was a closely related group of persistent CC6 isolates with a mean SNP distance of 11 collected between 2011 and 2013 and none of these isolates contained a QAC efflux gene. Yet, from 2011 to 2012 in this same facility there was a persistent group of CC199 isolates which all contained *bcrABC*. Also in this facility was a group of persistent CC155 isolates collected between 2011 and 2017 with a mean SNP distance of 10.8 which primarily contained *bcrABC*, but there were also isolates from this group in 2011 and 2013 which did not contain *bcrABC*. These differing patterns of QAC efflux gene prevalence among persistent *Lm* isolates within the same facility suggest a limited direct role for QAC efflux genes as predictors of persistence. Overall, our analysis of these nine facilities did not indicate a strong association between QAC efflux genes and long-term environmental persistence. This may be because QAC efflux genes in *Lm* only minimally increase the MIC of QAC-based sanitizers like benzalkonium chloride (BC). Some studies found that *bcrABC* and *qacH* only increased the MIC of BC to between 5 and 15 ppm from < 5 ppm [7, 61]. This level of reduced susceptibility is much lower than working sanitizer concentrations used in the food industry (200–1000 ppm), thus it may only be relevant in difficult to clean harborage points where sanitizer concentration may be diluted or contact time may be insufficient.

## Conclusion

We determined that *bcrABC* was widely distributed among *Lm* isolated from U.S. food processors. The prevalence of QAC efflux genes was significantly different among clonal complexes and was associated with stress adapted genotypes. Regional differences were observed in comparison to other international studies. For example, the stress adapted clonal complex CC321 was more common in the US and Canada compared to Europe. By contrast, CC121, another major stress adapted clonal complex, was more common in Europe compared to the US and Canada. This may also explain why isolates from these regions had different rates of *bcrABC* and *qacH* carriage.

Empirical, case-controlled studies testing the persistence of *Lm* with different genetic backgrounds in commercial food plants is not possible. However, in our observational study we did not find evidence of a strong association between QAC efflux genes and persistence. This suggests that other confounding factors besides QAC efflux genes complicate persistence. Confounding factors could include the presence of other genetic determinants that contribute to environmental persistence. Confounding factors could also be non-genetic, and rather related to the hygienic design and surrounding environment of the food processing facility. The presence of harborage points that are difficult to clean consequently limit sanitizer access. In this scenario, QAC efflux genes may provide a benefit for *Lm* established in specific harborage points that are only exposed to suboptimal concentrations of QAC based sanitizer. Future studies should investigate the effect of both genetic and non-genetic factors on persistence in *Lm,* such as the biophysical aspects of niche formation and how, if at all, QAC efflux genes and other stress genes affect survival in these environments.

## Supplementary Information

**Additional file 1.** Listeria Isolate Dataset. A list of isolates used in the analysis within this paper and their associated metadata. The SRR codes for each isolate are given which can be used to download the sequencing reads from NCBI.

## Data Availability

All isolate sequence data is stored on the NCBI Sequence Read Archive with corresponding ID codes. ID codes for all isolates used in this study are listed in Additional file 1.

## Abbreviations

QAC: Quaternary ammonium compound
*Lm*: *Listeria monocytogenes*
CC: Clonal complex
BC: Benzalkonium chloride
GWAS: Genome Wide Association Study
BLAST: Basic Local Alignment Search Tool
